# Menadione Sodium Bisulfite Loaded Rhamnolipid Based Solid Lipid Nanoparticle as Skin Lightener Formulation: A Green Production Beside In Vitro/In Vivo Safety Index Evaluation

**DOI:** 10.34172/apb.2024.047

**Published:** 2024-06-22

**Authors:** Fatemeh Asadpour Panbehchouleh, Hossein Amani, Majid Saeedi

**Affiliations:** ^1^Department of Biotechnology, Faculty of Chemical Engineering, Babol Noshirvani University of Technology, Babol, Iran.; ^2^Pharmaceutical Sciences Research Centre, Hemoglobinopathy Institute, Mazandaran University of Medical Sciences, Sari, Iran.; ^3^Department of Pharmaceutics, Faculty of Pharmacy, Mazandaran University of Medical Sciences, Sari, Iran.

**Keywords:** Anti-pigmentation effect, Dermal delivery, Menadione sodium bisulfite, Rhamnolipid, Solid lipid nanoparticles

## Abstract

**Purpose:**

In the current investigation, an ultrasonic approach was performed to produce menadione sodium bisulfite-loaded solid lipid nanoparticles (MSB-SLNs) with rhamnolipid as bio-surfactant, which aimed to increase the dermal delivery and anti-pigmentation effect.

**Methods:**

To achieve optimum delivery for MSB, the impact of the ratio of two surfactants (rhamnolipid: Tween) on nanoparticle attributes and the respective functions were evaluated. In vitro diffusion process, in vitro cytotoxicity assay, determination of melanin content of melanoma cells, L-DOPA auto-oxidation inhibitory test, and skin irritation studies carried out to investigate the suitability of MSB formulation in dermal application.

**Results:**

The optimized nanoparticles showed an average particle size, zeta potential, polydispersity index (PDI), and drug entrapment efficiency of 117.26±1.12 nm, -6.28±0.33 mV, 0.262±0.002, 83.34±0.75% respectively in hydrophilic-lipophilic balance (HLB) of 12. The in vitro diffusion process demonstrated that MSB-SLN gel had a prolonged release pattern. The levels of MSB in the cutaneous layers (52.192±2.730% or 961.59±50.313 μg/cm^2^ ) and the receiver compartment (23.721±1.803 % or 437.049± 33.236 μg/cm^2^ ) for the MSB-SLN gel was higher than MSB simple and showed no cutaneous irritancy and toxicity in rats. MSB-SLN inhibited melanin formation and was remarkably higher than free MSB. MSB-SLN inhibited L-3,4- dihydroxyphenylalanine (L-DOPA) auto-oxidation to a greater extent (95.14±1.46%) than MSB solution (72.28±0.83%).

**Conclusion:**

This study’s observations revealed that the produced MSB-SLN might be used as a potential nano-vehicle for MSB dermal administration, thereby opening up innovative options for the management of hyper-melanogenesis problems.

## Introduction

 A global class of biological pigments is melanin.^1^ Various enzymes in a complicated chemical procedure synthesize melanin in epidermal melanocytes.^2^ The most important enzyme in the stage of synthesizing melanin is tyrosinase.^3^ The most significant grievances of the skin include freckles and melasma, which are pigmentation disturbances generated via the unnatural distribution of melanocytes.^4^ Therefore, this irregularity in pigmentation can lead to psychological and social problems in patients.^5^ A series of skin lighteners are considered to treat hyperpigmentation disturbance because they can prevent melanin synthesis.^6^ Menadione sodium bisulfite (MSB)—one of the derivatives of menadione (vitamin K_3_)—can considered as a skin-lightener agent due to its ability to inhibit the synthesis of melanin as a result of decreased tyrosinase activity.^7^ Expansion of a delivery system for MSB that is a hydrophilic drug is so considerable, there are many challenges because of (*a*) having an undesirable half-life in the biological liquid (*b*) low permeability through barriers and unsuitable biodistribution, (*c*) low bioavailability.^8,9^ Due to characteristics like drug stability, small particle size, prevention of the degradation of the entrapped drug, controlled drug release, ease of manufacturing and scale-up, and increasing penetration of drugs through the skin due to their high affinity with the stratum corneum, solid lipid nanoparticles (SLNs) have been suggested as suitable drug delivery systems among all lipidic carriers.^10^ Therefore, for skin-lightening agents SLNs could be an appropriate drug delivery system. One of the essential components of SLNs is surfactant and synthetic surfactant is usually used for the preparation of SLNs.^11^ However, most synthetic surfactants illustrate detrimental effects on human health and the environment due to their toxic and corrosive nature.^12^ There is a great interest in biosurfactants due to their low toxicity, environmentally friendly, biodegradability, and functionality under extreme conditions.^13^ Rhamnolipids are one of the most important biosurfactants, mainly produced by *P. aeruginosa* and composed of rhamnose sugars attached to ß-hydroxy fatty acid chains.^14^ The interfacial activity of rhamnolipids has been utilized for bioremediation of polluted soils,^15^ also they can be used as pharmaceutical ingredients due to their antimicrobial and anticancer activity.^16,17^ No research has investigated the use of rhamnolipid biosurfactants in the formulation of SLN containing MSB. In addition, ultrasound as a green technique was used for the preparation of MSB-SLN. The anti-melanogenesis effect and the efficacy of binary surfactants in the creation of MSB-SLNs were evaluated by none of the studies. Also, there isn’t any investigation about the quantity of melanin and inhibition impact of SLNs including MSB on auto-oxidation of L-DOPA. In addition, the irritation effect of MSB-SLN on the skin has not been carried out by any research. An ultrasonic method was utilized to make MSB-SLN gel for more investigation on the characterization above-mentioned and skin cells were used for in vitro investigation of cellular safety. Therefore, the encapsulation of MSB in SLN shows that SLN as a suitable carrier can be replaced with the old carriers, and also MSB-SNL gel can be developed for topical administration as a cosmetic product.

## Materials and Methods

###  Materials

 MSB was purchased from Osve Pharmaceutical Company (Tehran, Iran). Beeswax (BW) was obtained from John’s laboratory (John’s Laboratory Chemicals, India). Tween 80 was purchased from Merck (Merck Co., Germany). Distilled water was treated by a Human Power 2 system (human Co., Korea). L-3,4- dihydroxyphenylalanine (L-DOPA), 3(4,5 di-methylthiazol-2-yl)-2,5 diphenyl-tetrazolium bromide (MTT), and DMSO, were obtained from Sigma Chemical Co. (St. Louis, MO, USA).

###  Microorganism

*Pseudomonas aeruginosa* ATCC 27853 was purchased from the Darvash Company, Tehran, Iran.

###  Preparation of rhamnolipid

 Preparation of pre-cultivation is the first step in the production of rhamnolipid and Lactose broth (LB) was utilized for this purpose. In a 250-mL Erlenmeyer flask, 100 mL LB medium at 121 °C for 15 minutes was autoclaved, and then a loop of the bacterium was inoculated into the above medium under a sterile condition (pre-culture). This flask was incubated in an incubator shaker (Mehr Tajhiz, Iran) at 37 °C and 150 rpm for 24 hours.^18^ A production medium consists of a salt solution containing 1 g/L KCl, 6.27 g/L Na_2_HPO_4_.2H_2_O, 0.5 g/L MgSO_4_.7H_2_O, 10.11 g/L NaH_2_PO_4_.2H_2_O, 15 g/L NaNO_3_; 0.1 M sodium phosphate buffer at pH 6.5 and 125 g/L sunflower oil was utilized for the culture all over the study. A trace element solution (1 mL) containing: 0.8 g/L MnSO_4_.H_2_O, 1.2 g/L CoCl_2_.6H_2_O, 2 g/L trisodium citrate.2H_2_O, 1.4 g/L ZnSO_4_.7H_2_O, 1.2 g/L CuSO_4_.5H_2_O, 0.28 g/L FeCl_3_.6H_2_O was sterilized via a filtration procedure (0.22-μm) since it is sensitive to heat and then added to the medium. Also, 10 mL of the pre-culture was incubated in the Erlenmeyer flask. Finally, 200 mL of the production medium (above-mentioned) in a 500 mL Erlenmeyer flask at 37 °C for 7 days was placed in an incubator shaker at 150 rpm.^19^ After 7 days of cultivation, the extraction of rhamnolipid was done according to Müller et al.^20^ First, centrifugation of the culture broth with the n-hexane 1:1 (v/v) at 6000 rpm was done for 15 min to separate the n-hexane phase, aqueous phase (supernatant), and cells. Then, 85% phosphoric acid 1:100 (v/v) was used to acidify the aqueous phase and adjust the pH in the range of 2-3. Afterward, by adding an equal amount of ethyl acetate to the acidified supernatant and centrifugation, rhamnolipid entered the ethyl acetate phase. Finally, ethyl acetate was evaporated in a rotary evaporator, and pure rhamnolipid was obtained.

###  Characterization of the produced rhamnolipid by ATR-FTIR

 A Cary 630 FTIR spectrometer (Agilent Technologies Inc., CA; USA) was applied to identify the chemical structure and the functional groups of the produced rhamnolipid in attenuated total reflectance (ATR) mode. At a resolution of 2 cm^-1^ from 4000-650 cm^-1^ the spectra were recorded.^18^

###  Preparation of the calibration curve

 After preparing different concentrations of the drug (menadione sodium bisulfite) in the appropriate solvent (ultra-pure water), the absorbance numbers were calculated using a UV spectrophotometer (Photonix Ar, Iran). Finally, a standard curve was drawn based on these two parameters.

###  Production of MSB-SLNs

 The ultrasonication procedure was applied to produce MSB-SLNs.^21^ To obtain an oil phase, a combination of rhamnolipid and beeswax was heated up to 75 °C with a heater stirrer. Then, Tween 80 and menadione sodium bisulfite were dissolved in deionized water to obtain an aqueous phase. This aqueous phase was homogenized using an ultra-homogenizer and then heated up to 75 °C. After achieving the demanded temperature, to make a pre-emulsion the aqueous phase was appended to the oil phase in a dropwise manner under the agitation condition. Finally, sonication of the blend was carried out for 3 minutes through a probe sonicator (Bandelin, 3100, Germany). To achieve MSB-SLNs, the nanoemulsion was kept in an ice bath for 30 minutes.

###  Production of MSB-SLN gel and MSB simple gel

 By dispersion of carbopol 1% in water, retaining it in the refrigerator for 24 hours followed by neutralization of the carbopol solution by adding triethanolamine (in a dropwise manner with stirring), the plain gel was prepared. Preparing the MSB-SLN gel was carried out by a combination of 10 g of simple gel with 10 g of MSB-SLN (200 mg MSB) at 400 rpm utilizing a propeller homogenizer. Moreover, the combination of 10 g of simple gel with 10 g of MSB-solution at 400 rpm utilizing a propeller homogenizer was performed to make MSB simple gel.

###  Physicochemical characterization 

 Using Zeta Sizer Nano (Malvern Instruments, Worcestershire, UK), polydispersity index (PDI), zeta potential, and the mean particle size of MSB-SLNs with an angle of 90° at 25 °C were measured by dynamic light scattering (DLS) method.^22^ All measurements have been done in triplicate.

###  Entrapment efficiency 

 The MSB-SLNs were exposed to centrifugation at 27000 rpm (Sigma, Germany) and 4°C for 30 minutes to separate loaded MSB from the dispersion. A syringe filter (0.22 μm) was utilized for the filtration of supernatant and then with a spectrophotometer at 264 nm the MSB content in the filtered solution (free drug) was specified. Utilization of the slope (S) of the calibration curve and the standard deviation (SD), the quantification limit (LOQ) and detection limit (LOD) were calculated in this way: LOD: 3.3 SD/S, LOQ: 10 SD/S.^21^ The amounts of LOD and LOQ were 0.343 and 1.041 µg/mL respectively. To calculate drug entrapment efficiency equation 1 was utilized.


Eq. (1) $$EE\%=\frac{W_{total}-W_{free\;drug}}{W_{total}}\times 100$$

W _total_ = Total quantity of MSB utilized in the formulation

 W _free drug_ = The quantity of drug found in supernatant after centrifugation of the MSB-SLNs formulation.^23^

###  Scanning electron microscopy (SEM)

 The morphological study of MSB-SLN was carried out through SEM. After covering the formulation with gold in the DSR1 nano construction covering, the observation was done at an acceleration voltage of 20.0 kV through SNE_4500M SEM at a magnification of 10000 X.^24^

###  ATR-FTIR spectroscopic analysis

 Investigation of the possible interactions of the formulation components was carried out via a Cary 630 FTIR spectrometer (Agilent Technologies Inc., CA; USA) with a diamond ATR. To this end, SLN was first freeze-dried. Then ATR-FTIR examination of this sample, as well as other components that generate SLN including beeswax, Tween 80, MSB, and rhamnolipid was accomplished in the scan scope of 4000-650 cm^-1^ and a resolution of 2 cm^-1^.^24^

###  Differential scanning calorimetry (DSC)

 Thermal behavior of the pure drug, lipid, and drug-loaded SLNs was studied via DSC (Pyris 6, PerkinElmer, USA). Five milligrams of freeze-dried above-mentioned samples were placed in a DSC pan separately. The samples were examined by a heating rate of 10 °C/min and a temperature scope of 30-300 °C under N_2_ atmosphere.^25^

###  In vitro drug release 

 To detect the effectiveness of SLNs, one of the most important parameters is the drug’s release profile.^26^ Through the paddle technique (kind II) noted in United States Pharmacopoeia (USP XXVIII, 2005), the release profile of MSB-SLNs was determined. To perform the drug release experiments, the Erweka dissolution apparatus (DT620, Erweka, Germany) was employed. The dissolution medium (900 mL) at 37 °C was prepared for this experiment and the rotation speed was 100 rpm. Sampling of the dissolution medium (water in this case) was done at specific time intervals (2, 4, 6, 8, and 24 hours) and was replaced with fresh water at the same temperature. The quantity of MSB in the samples was specified via the UV spectroscopy at 264 nm.^2,22,27^

###  In vitro skin permeation study 

 All research on animals is carried out in agreement with the ARRIVE principle and follows UK guidelines (Scientific Procedures). Franz diffusion cell was used for in vitro skin absorption examination by an effectual diffusional area (3.8 cm^2^). Therefore, through 13 mg xylazine/kg and 87 mg ketamine/kg of body weight, the male Wistar rats (with a weighting of 120-150 g) were anesthetized and then shaved. 48 hours later, chloroform inhalation was employed to scarify the rats, and then, removing the abdomen skin of rats was carried out by surgery. After removing the adherent subcutaneous fats and before starting the diffusion test, these skins were placed for 24 hours in a normal saline solution. The stratum corneum and the dermal part of the skins were put into the Franz diffusion cell in a way that faced the donor and receptor compartment respectively. Deionized water was used to fill the receiver part and a jacket containing thermostatically controlled water circulation near the cell body was used to maintain the diffusion cells at 32 °C throughout the tests. On the donor section (dorsal part of rats’ skin) one gram of the control and the sample (MSB simple gel and MSB-SLN gel respectively) were spread uniformly. Samples were taken at predetermined time intervals (2, 4, 6, 8, 10, and 24 hours) from the receptor chambers and replaced with fresh deionized water in an equivalent volume. All samples were analyzed using a UV spectrophotometer at 264 nm. When the examination was over, to determine the quantity of precipitated MSB in the skin, the skins were separated, and then deionized water was applied to rinse them three times. The skins were dried and cut into little parts and kept in water tubes for 24 hours, and then were sonicated for 1 hour using a bath sonicator. Filtration of the supernatant was carried out through a filter paper; next a syringe filter (0.22-μm) was utilized. To determine the quantity of the samples, the UV spectrophotometer at 264 nm was used.^21,28^

###  Cell viability investigation

 The National Cell Bank (Pasteur Institute of Iran; Tehran, Iran) provided human foreskin fibroblast (HFF) cells to evaluate the capability of the formulations for in vitro cell survival. Seeding the cells was carried out using the various concentrations of SLN containing MSB, blank hydrogel, and pure MSB (400, 200, 100, 50, 25, and 12.5 µM) or the carrier control for 24 h in the base of microplates (Nunclon) at a mass of 10^5^ cells. Earlier than taking away the substances, the cells were rinsed with phosphate-buffered saline (PBS) and the cell survival was evaluated by colorimetry formazan (MTT). Incubation of the cells was carried out at 37 °C for 4 h by addition of 0.5 mg/mL of the MTT. Before dissolving the silt in dimethyl sulfoxide (DMSO) (100µL) containing formazan crystals, the supernatant was taken away. In addition, 20 min after shaking the plates, a multi-walled spectrophotometer was applied to determine the optical density at 560 nm. Different doses (12.5, 25, 50,100, 200, and 400 µM) in triplicate were used to estimate the cell survival with six extra controls, and the cells’ vitality was calculated by equation 2.^27^


Eq. (2) $$\%Cell\;survival=\frac{Absorbance\;Sample-Absorbance\;Blank}{Absorbance\;Control-Absorbance\;Blank}$$

###  Determination of melanin quantity of melanoma cells

 To specify the quantity of melanin, melanoma cells of Murine B16F10 obtained from the Pasteur Institute of Iran and were seeded in a 12-well plate overnight in RPMI media containing penicillin: 100 U/mL, fetal bovine serum: 10% (FBS; Gibco, USA), and streptomycin: 0.1 mg/mL. After 24 hours, treating the cells was carried out by MSB-SLN and MSB (2 mM) and incubation was done for 24 hours. At 100 °C for 30 minutes, the cell pellets were dissolved in NaOH (2 M) (100 μL), and then the comparison of melanin content with the control was performed by Microplate Reader (Bio-Tek, Winooski, USA) through their absorbance at 405 nm.^29^

###  L-DOPA auto-oxidation inhibitory test

 The inhibition effect of formulation and pure MSB on auto-oxidation of L-DOPA was investigated according to Hwang and Lee.^29^ Preparation of various concentrations of SLN containing MSB and MSB in sodium phosphate buffer (0.1 M) (pH = 6.5) was carried out in a 96-well plate, and then 10 mM L-DOPA at pH 6.5 (in sodium phosphate buffer 0.1 M) was transferred to these 96–well plate. Finally, incubation of this blend was carried out at 37 °C for 15 minutes. The quantification of activity was performed at 475 nm.

###  Skin irritation evaluations

 In this research, the protocols of the Institute Animal Ethical Committee were used. To perform a skin irritation experiment, the dorsal part of Wistar rats was shaved 24 h before the experiment.^30^ In this way, the animals were divided into groups of six (5 groups). Group I was considered as control and groups II, III, IV, and V (typical irritant) received MSB-SLN hydrogel (optimum hydrogel), simple MSB hydrogel, free drug hydrogel, and formalin aqueous solution 0.8% (v/v) respectively.^31^ Using the above-mentioned formulations for three sequential days on the skin of rats, a visual evaluation of the tested area was performed.

###  Statistical analysis

 To analyze the results of this research work, GraphPad Prism 8 was utilized and the expression of the results was carried out as mean ± standard deviation. On the other hand, statistical analysis of defined variables was done through Tukey test LSD’s post-hoc test and analysis of variance. *P* < 0.05 was regarded as statistically significant (n = 3).

## Results and Discussion

###  Confirmation of rhamnolipid production by ATR-FTIR analysis

**Figure 1:**
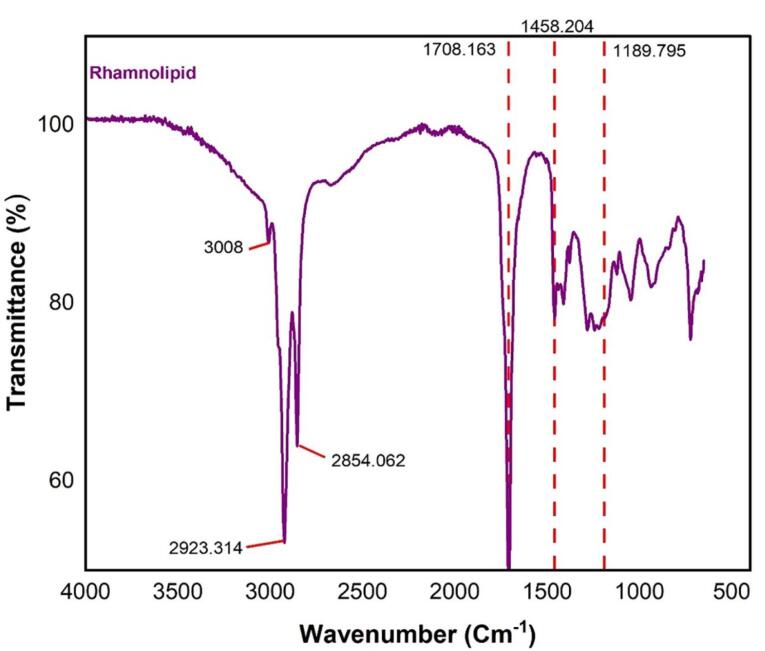
ATR-FTIR analysis of produced rhamnolipid of *P. aeruginosa* ATCC 27853

 To confirm the production of rhamnolipid using *P. aeruginosa* ATCC 27853, FTIR analysis was performed. The result of the FTIR experiment is shown in Figure 1. According to Figure 1, the existence of long-chain hydrocarbon and rhamnose sugar was established through FTIR analysis. Stretching (C-H) of CH_2_ and CH_3_ groups are represented by absorption at 2923 cm^-1^ and 2854 cm^-1^. Also, an absorption at 1708 cm^-1^ represents a carbonyl stretching band (C = O). Moreover, absorption at 1458 cm^-1^ and 1189 cm^-1^ respectively represent stretching bands of (C = C) and (C-O). Consequently, the glycolipid structure of rhamnolipid produced by *P. aeruginosa* ATCC 27853 was proved by comparing the main peaks of rhamnolipid with the results of the research conducted by Leitermann et al^32^ and Rikalovic et al.^33^

###  MSB-SLN characteristics analysis

**Figure 2:**
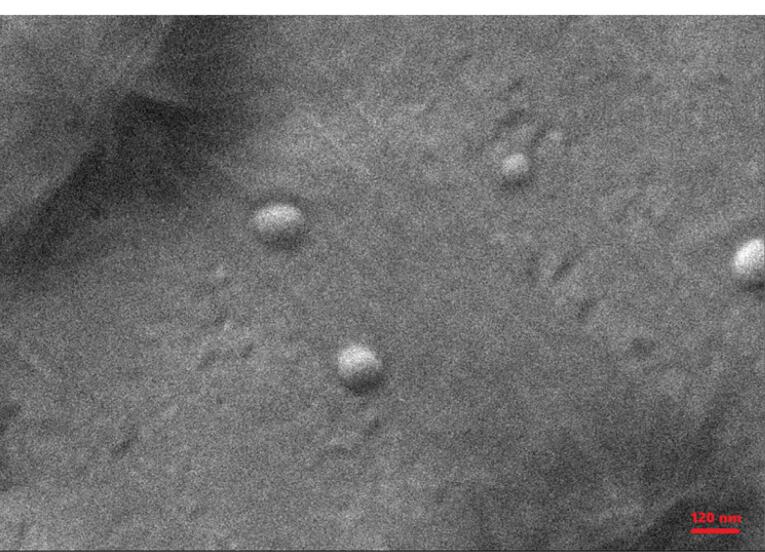
SEM picture of MSB-SLN

 SLN containing MSB was produced using the ultrasonication method described in the Materials and Methods section. Optimization of formulations was done in the range of hydrophilic-lipophilic balance (HLB) values obtained as a result of using different ratios of rhamnolipid biosurfactant and Tween 80 surfactant. The average particle size, which is a significant parameter to ensure the efficiency of the system, as well as the particle size distribution (PDI) which shows dispersion quality have been investigated in this research. The results of these two parameters (particle size and PDI) and the compositions of prepared SLNs are shown in Table 1. Previous studies showed that the PDI value is commonly between 0 and 1. On the other hand, the breadth of particle size distribution was demonstrated in PDI values higher than 0.7.^34^ Based on this, the PDI value in this research (less than 0.6) demonstrated a satisfactory distribution for formulations prepared with a combination of surfactants. By comparing F1 with F2-6 formulations (according to Table 1), it was found that the particle size and PDI for all formulations containing the double combination of surfactants have low values and the change in the ratio of surfactants led to a change in these two parameters. It was found that by setting the HLB to 12 (and bringing it to the lipid HLB) by adding a co-emulsifier (rhamnolipid), the particle size considerably decreases from158.833 ± 1.193 nm to 117.266 ± 1.123 nm (*P* < 0.05), which can prove superior stabilization of the formed emulsion in the presence of rhamnolipid (the second surfactant). On the other hand, the dispersion of surfactants in the aqueous and oil phases with great and small HLB, respectively, results in greater stabilization of the surfactant layer at the interface.^28^ Therefore, it can be concluded that the use of a combination of surfactants leads to an improved stability of the formed emulsion. Table 1 shows that the zeta potential values are from -0.619 mV to -6.83 mV. The reason for the negative zeta potential of the prepared formulations (while the drug particles possess a positive charge) is known based on previous studies, which state ethoxy groups in non-ionic surfactants that have a dipolar nature are responsible for creating a negative zeta potential surrounding the colloidal particles.^23^ Encapsulation in SLN by the creation of a surface coating and reduction of electrophoretic mobility can decrease the zeta potential and subsequently enhance the stability of the formulations. Still, this research work used Tween 80 and rhamnolipid surfactants to create higher steric stabilization.^23^ Due to the creation of condensed barriers surrounding the colloidal particles as a result of using the double combination of surfactants, more negative charge is created around the particles. Also, reducing the proportion of surfactants from 1:0 to 1:1.68 increased the total zeta potential of the formulations from -0.619 ± 0.090 mV (F1) to -6.28 ± 0.33 mV (F4) (*P* < 0.05). According to the findings, it can be said that the ratio of Tween to rhamnolipid considerably impacts the zeta potential and also controls the particle size. The greatest zeta potential was also acquired in the closest ratio of surfactants to each other (Table 1). It was also found that the zeta potential of the formulations is affected by both the particle size and the ratio of surfactants. Therefore, nanoparticles with greater negative charges can be formed as a result of carrying larger amounts of surfactants around small nanoparticles. Non-ionic surfactants can create complete coverage on the surface of the particles,^34^ but this ability may be lost due to the superior concentration of the other surfactant in cases where the combination of two surfactants is used.^22^ So, it causes a decrease in the zeta potential, and this is the reason for the low zeta potential of F6 compared to F4 (decreased surfactant concentration around nanoparticles) regardless of particle size. On the other hand, in the dispersion medium, the Tween 80 monomers concentration must be small due to the small concentration of critical micelles (about 0.015 mM).^22^ Otherwise, the possibility of surfactant monomers being absorbed into hydrophobic lipid surfaces increases instead of forming micelles. In addition to all this, it should be noted that zeta potential was not taken into account as the main criterion in this research for choosing the optimal formulation. The zeta potential of MSB-SLNs was re-measured at 10, 30, and 60 days. The results showed no significant changes. Also, a visual study showed during the production process of SLNs and doing experiments for two months, no changes in the physical appearance of the formed emulsion such as precipitation or accumulation of particles were observed, which proves the stability of the drug. Meanwhile, for more confirmation, SEM analysis was performed after 60 days. As you can see in Figure 2, the size of the formed nanoparticles has not changed much compared to the first day, which was 117 nm (Table 1). Therefore, it can be concluded that the formed MSB-SLN is stable. The range of EE% is from 61.08 ± 0.99% % to 83.34 ± 0.75% and the results are shown in Table 1. Several factors including the polymorphic state of the lipid phase, the solubility and miscibility of the drug in the lipid matrix, can impact the EE%.^35^ On the other hand, the excretion of the drug increases with the increase of the specific surface area (as a result of the reduction of the particle size), which causes an EE% decrease.^22,36^ In addition, the great inclination for the escaping of the drug from the matrix of lipids is due to its great solubility in water. So, to overcome this problem and increase EE capacity, the crystalline state of the lipid can be reduced (as a result of the reduction in the concentration of beeswax). The reason for low entrapment efficiency could be due to the high HLB of the system, and if the amount of rhamnolipid enhances, the low HLB can help to load MSB into the solid lipid matrix.^37^ Although the solubility of MSB in water (55 mg/mL at 25 °C) is much greater than the solubility of gentamicin^38^ and ciprofloxacin hydrochloride^39^ (50 and 35 mg/mL respectively), which were examined in previous studies, however, the EE% of MSB in SLN was much greater compared to the EE% of the drugs mentioned above. Considering the results of the particle size, zeta potential, and specifically, the EE% F4 was chosen as the optimal formula for further research.

**Table 1:** Component and physicochemical properties of investigated MSB- SLN

**Code **	**Beeswax (mg)**	**MSB (mg)**	**Tween 80 (mg)**	**Rhamnolipid (mg)**	**Particle size (nm)**	**ZP (mv)**	**PDI**	**EE (%)**	**HLB**
F1	100	200	250	0	158.83 ± 1.19	-0.61 ± 0.09	0.256 ± 0.012	61.08 ± 0.98	15
F2	100	200	230	20	101.30 ± 1.25	-5.84 ± 0.68	0.281 ± 0.003	62.61 ± 2.46	14.6
F3	100	200	140	110	105.86 ± 1.85	-4.38 ± 0.50	0.276 ± 0.002	69.503 ± 0.89	12.6
F4	100	200	110	130	117.26 ± 1.12	-6.28 ± 0.33	0.262 ± 0.002	83.34 ± 0.75	12
F5	100	200	93	157	138.90 ± 1.50	-6.83 ± 0.27	0.301 ± 0.002	78.15 ± 1.18	11.6
F6	100	200	48	202	152.86 ± 1.72	-3.25 ± 0.44	0.285 ± 0.003	68.20 ± 1.21	10.6

The data are the mean and standard deviation of three determinations (n = 3). Menadione sodium bisulfite: MSB; ZP: zeta potential; PDI: polydispersity index; EE: entrapment efficiency.

###  SEM analysis

 Morphological study of the optimized formulation carried out through SEM. SEM picture for MSB-SLN (Figure 2) demonstrated that the particles were uniform in size, spherical in shape, and smooth in surface.

###  ATR-FTIR analysis

**Figure 3:**
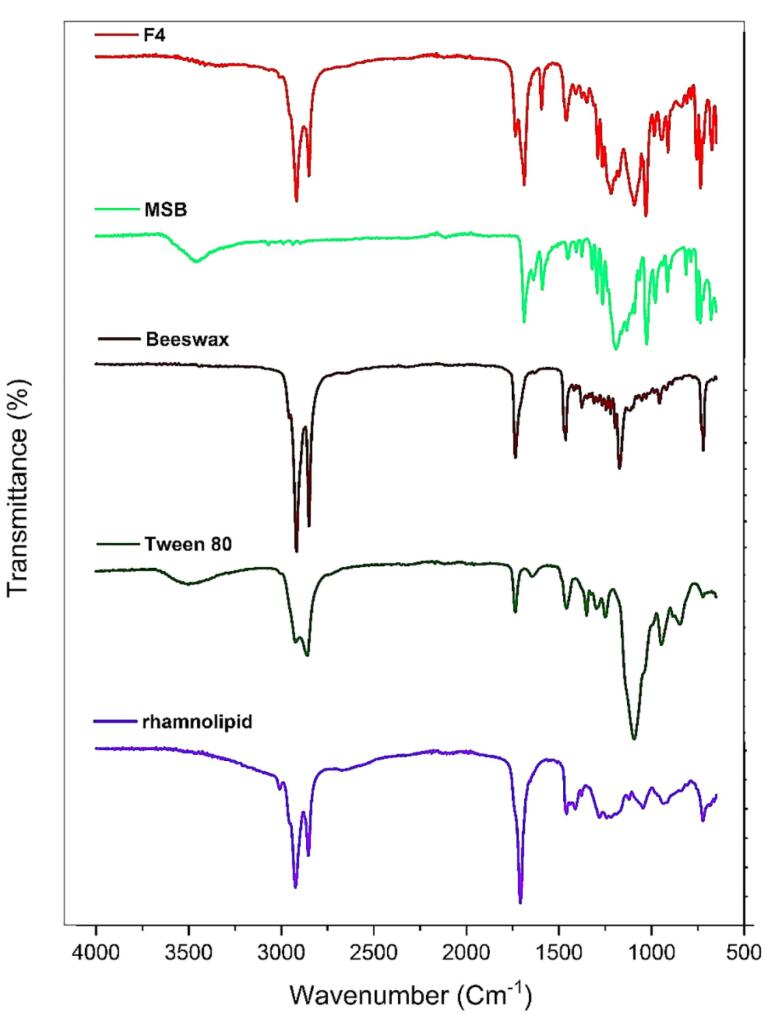
ATR-FTIR spectra of F4, MSB, Beeswax, Tween80, and rhamnolipid

 Investigation of the chemical interaction of the components used to generate SLNs containing MSB was carried out using the ATR-FTIR method. The ATR-FTIR spectra of rhamnolipid, Tween 80, pure MSB, beeswax, and F4 are shown in Figure 3. In the ATR-FTIR spectrum of MSB the diagnostics peaks were observed at 1687 cm^-1^ (C = O, stretching), 1637 cm^-1^ (C = O, stretching), and 1589 cm^-1^ (C-C, stretching). Beeswax represented peaks at 2918 cm^-1^ (asymmetric stretching of -CH_2_-), 2849 cm^-1^ (symmetric stretching of -CH_2_-), 1733 cm^-1^ (stretching of C = O), 1463 cm^-1^ (bending of -CH_2_-), and 1171 cm^-1^ (stretching of C-O). Tween 80 demonstrated peaks at 3497 cm^-1^ (stretching of O-H), 2922 cm^-1^ (asymmetric stretching of -CH_2_-), 2858 cm^-1^ (symmetric stretching of -CH_2_- ), and 1735 cm^-1^ (stretching of C = O). The ATR-FTIR spectrum of rhamnolipid illustrated characteristic peaks at 3008 cm^-1^ (symmetric stretching of O-H), 2923 cm^-1^ (asymmetric stretching of C-H), 2854 cm^-1^ (symmetric stretching of C-H), 1708 cm^-1^ (stretching of C = O), 1458 cm^-1^ (C–H, deformations), 1189 cm^-1^ (C–O, stretching). According to the results of ATR-FTIR, as the main diagnostic peaks for MSB were observed in the FTIR of formulation F4, there wasn’t any chemical interaction among compounds used to generate SLNs and drugs in the F4 formulation.

###  DSC analysis

**Figure 4:**
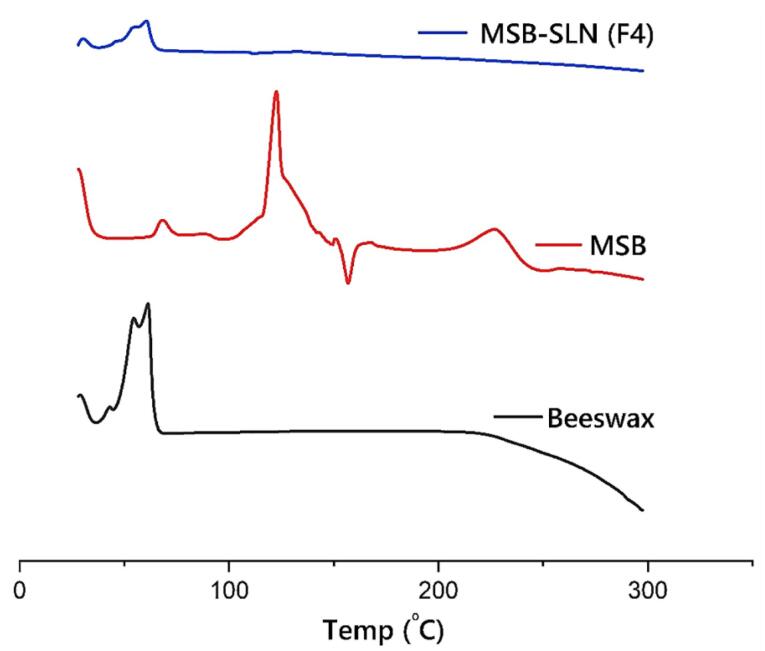
DSC traces of MSB, Beeswax, and MSB-SLN (F4)

 The DSC traces of beeswax, pure MSB, and MSB-SLN are demonstrated in Figure 4. The endothermic peaks at 122.5 °C and 40-66 °C demonstrated at MSB and beeswax thermograms respectively. The highly crystalline properties of MSB and beeswax were indicated by these peaks, which are at their melting point. The MSB endothermic peak vanished in the DSC thermogram of F4. However, the endothermic peak of beeswax is existed near the melting point of F4. This could be due to the amorphous state of MSB in the SLN or molecular dispersion of MSB within the SLN.

###  Drug release study

**Figure 5:**
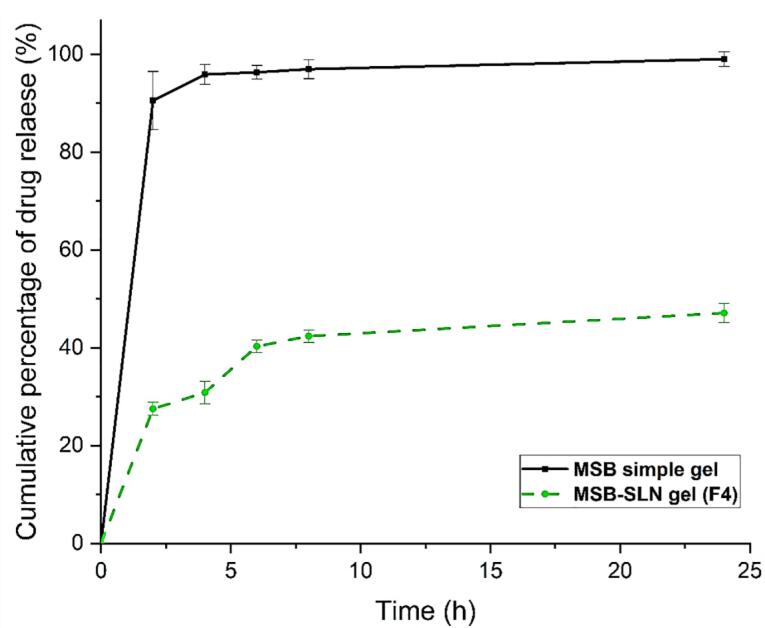
The release profile of MSB from MSB-SLN (F4) and simple MSB

 As reported in previous research, the bioavailability of the drug is related to controlling its release from SLN.^22^ In vitro release study was performed to investigate and make a comparison of the release behavior of the drug from MSB simple gel and MSB-SLN gel. The results of the in vitro drug release experiment (Figure 5) demonstrated that drug release from MSB-SLN gel was 12.612 ± 1.063% up to 2 hours, and a stable release existed up to 24 hours. Whereas in the case of MSB simple gel, in the first 8 h, 95% of the drug was released. As expressed in previous studies, the encapsulation of drugs in the lipid matrix of SLNs creates a targeted and controlled release of the drug as a result of reducing the mobility of the drug in the solid state (the advantage of using solid lipids compared to liquid lipids). The most significant parameters that impact the drug release rate from SLNs are viscosity within the matrix, high diffusion coefficient, molecular size, and high surface area.^40^ In addition, the speed of drug release depends on its encapsulation in the SLN matrix, so if the drug is loaded in the outer shell or inside the lipid core, it will be released quickly and slowly, respectively.^40,41^ To improve and control drug release from SLN formulations and increase the effectiveness of drug delivery systems, many studies have been conducted.^42,43^

###  In vitro percutaneous absorption study

**Figure 6:**
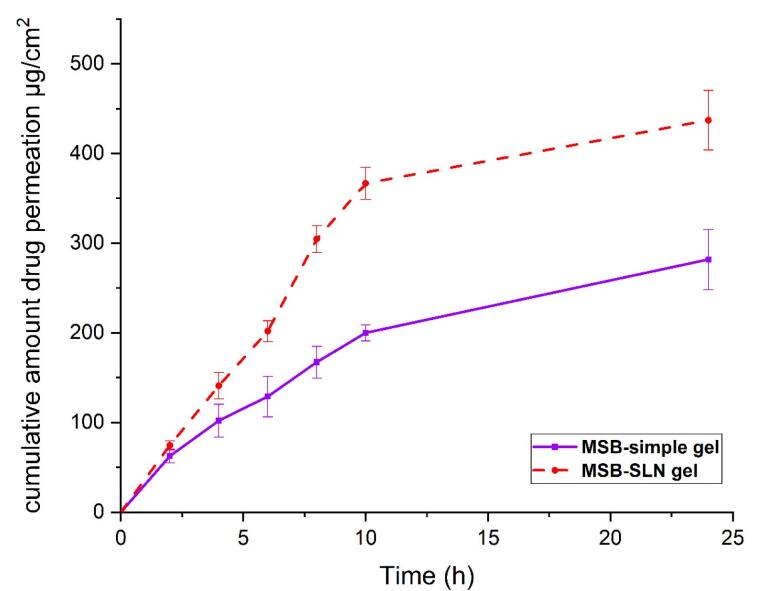
The cumulative amount of MSB permeated across rat skin (data are mean and standard deviation of three determinations, n = 3). The Sample represents MSB-SLN gel and the standard is MSB solution gel containing the same concentration of MSB

**Figure 7:**
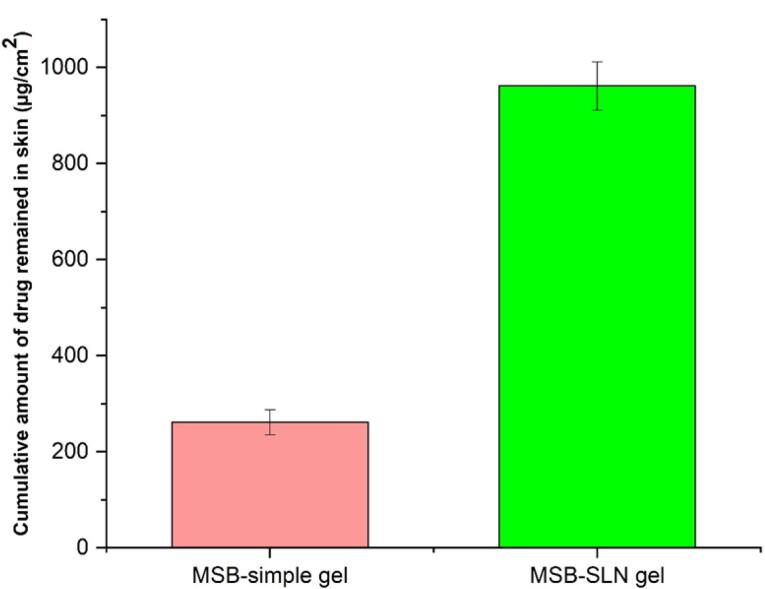
The cumulative amount of MSB remained in rat skin (error bars are standard deviation, the sample represents MSB-SLN and the standard formulation is the MSB solution gel containing the same concentration of MSB)

 Transdermal delivery (penetration of cumulative quantities of the drug via the skin of rat) and dermal delivery (penetration of the drug into the skin layer of rat) for the simple gel of MSB (as control) and MSB-SLN gel (F4) were measured using the in vitro skin absorption examination. Results were demonstrated in Figures 6 and 7. The MSB-SLN gel (F4) demonstrated a higher penetration into and across the cutaneous layers, revealing that it is highly appropriate for transdermal delivery compared to the MSB simple gel (p < 0.05). So, it could be said that MSB-SLN gel is suitable for transdermal delivery. In the receptor part, the maximum amount of MSB for the simple gel (15.299 ± 1.824% or 281.873 ± 33.607 μg/cm^2^) was much lower than that of the MSB-SLN gel (23.721 ± 1.803% or 437.049 ± 33.236 μg/cm^2^) (*P* < 0.05). In addition, the remaining content of MSB in the skin for the simple gel of MSB (14.210 ± 1.511% or 261.812 ± 26.087 μg/cm^2^) was significantly less than that for the MSB-SLN gel (52.192 ± 2.730% or 961.59 ± 50.313 μg/cm^2^) (*P* < 0.05). In addition, the carbopol polymer gelling properties are also very significant for drug penetration increase from formulations.^44^ Moreover, from previous studies, drug penetration in the skin is affected by the HLB value of SLN.^35^ Also, it has been demonstrated in previous research that the cumulative percentage of miconazole nitrate in the skin can be remarkably enhanced by miconazole nitrate–SLN gel in comparison to the marketed gel.^45^ However, based on the results of this study, it can be said that the most excellent substitute for ordinary gel in transdermal and dermal applications would be the SLN formulation.

###  MTT assay examination 

**Figure 8:**
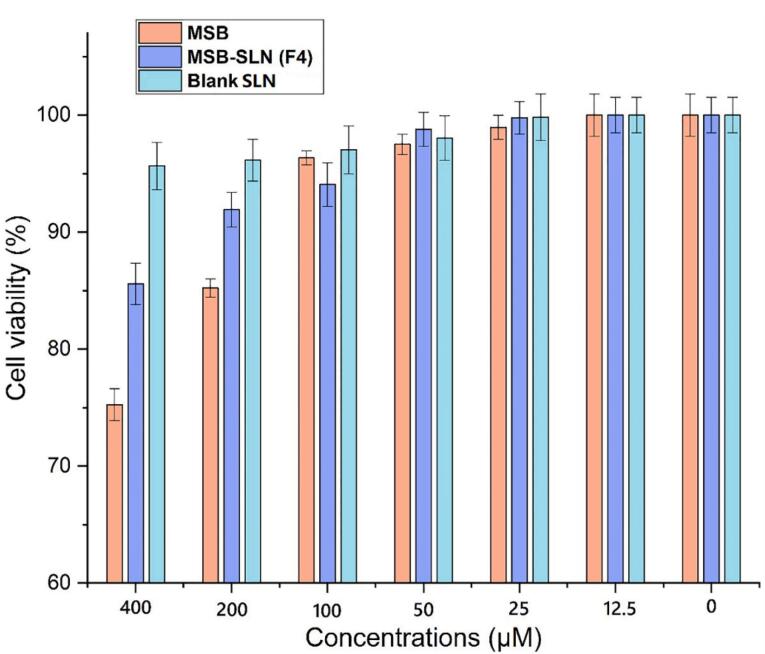
Viability of cell at diverse concentrations of MSB, MSB-hydrogel, and bare hydrogel (data are mean ± SD, n = 3)

 One of the simple techniques to measure the metabolic activity of living cells is to use MTT analysis. In this research work, the investigation of cell survival when various concentrations of MSB-SLN hydrogel, blank hydrogel, and MSB simple hydrogel were used at 12.5-400 µM carried out by MTT. Figure 8 shows the concentration-related cytotoxicity of HHF cells treated with MSB-SLN hydrogel, MSB simple hydrogel, and blank hydrogel in different concentrations for 24 hours. The percentage of cell survival after incubation with MSB-SLN and blank SLN for 24 hours in 400 µM was 85.58 ± 1.77 and 95.64 ± 2.01%, respectively, whereas with MSB simple hydrogel, it was 75.24 ± 1.35%. The results show when the treatment is carried out with MSB-SLN, the cell viability increases. This can be related to the ability of SLNs to control the release of the drug. So, a lower percentage of the drug is released and precipitates in the vicinity of the cell and thus causes less toxicity to the cell. In addition, one of the parameters affecting the cytotoxicity can be the surface charge of SLNs. As the optimal formulation (F4) has a low zeta potential (-6.28 ± 0.33 mV), it reduces the charge distribution surrounding the particles and subsequently their toxicity.^46^

###  Effect of MSB-SLN hydrogel on the synthesis of melanin

**Figure 9:**
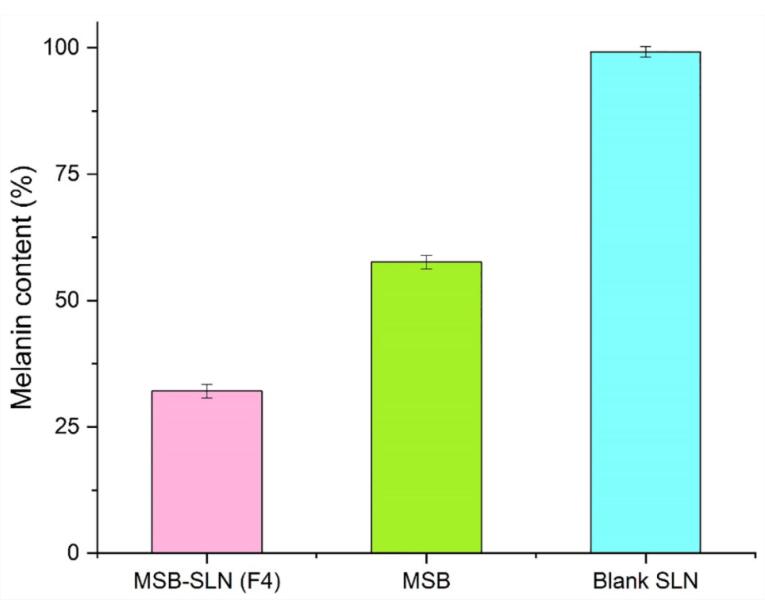
Melanin content of MSB-hydrogel, MSB, and blank hydrogel at 15μM concentration

 As stated in the previous sections of this study, irregularity in melanin synthesis causes skin problems. Since the tyrosinase enzyme is considered the most important enzyme in the production of melanin, in this way, the effect of MSB-SLN on reducing the activity of tyrosinase can ultimately reduce the amount of melanin present in the cells. In this research work, B16F10 cells were utilized to evaluate the effect of the prepared formulation on melanin synthesis. Figure 9 shows the results of this analysis, and it can be concluded that the inhibitory effect on melanin synthesis for simple MSB is lower than for MSB-SLN. Figure 9 reveals that MSB-SLN (F4) had the highest effect on reduction in melanin synthesis (32.09 ± 1.38 %) in comparison with MSB solution (57.61 ± 1.33 %) at 15µM concentration.

###  Inhibition of L-DOPA auto-oxidation assay

**Figure 10:**
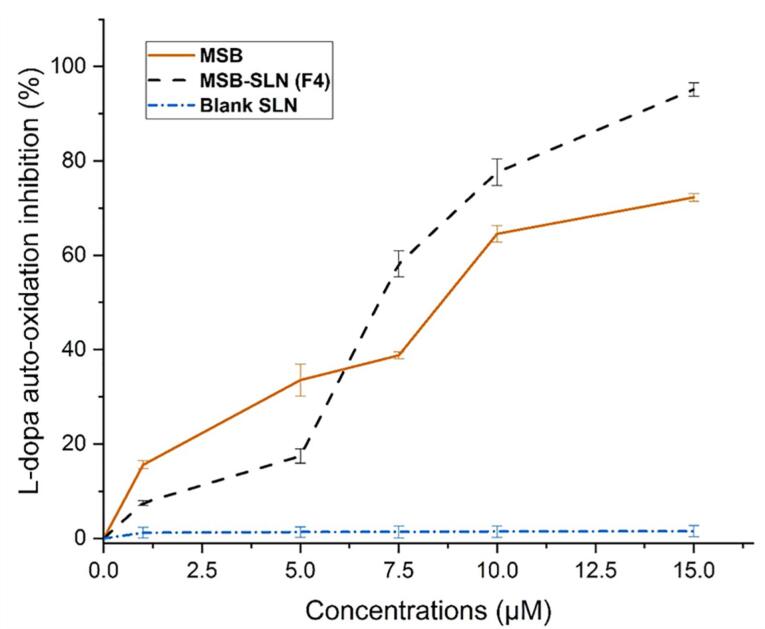
Inhibitory effect of MSB, MSB-SLN hydrogel, and blank hydrogel on L-DOPA auto-oxidation

 The most difficult challenge in dermatology and cosmetics is managing undesirable hyperpigmentation, and to overcome this challenge, it is necessary to create products with anti-melanogenic properties.^47^ According to the results, the auto-oxidation of L- DOPA in a concentration-related manner, was prevented by a simple MSB solution and MSB-SLN. At a concentration of 15 µM, the maximum percentage of inhibition (72.28 ± 0.83%) was obtained for the MSB solution. Based on Figure 10, an increase in SLN content increased the inhibition of L-DOPA (95.14 ± 1.46%). The long-term inhibition for MSB-SLN at concentrations of 1 to 5 μM can be related to the release behavior of MSB from SLN (Figure 5). In general, the inhibitory effect of MSB-SLN is significantly higher (IC50 value of 7.19 ± 0.28 µM) compared to pure MSB (IC_50_ value of 9.11 ± 0.41 µM) (*P* < 0.05). According to the results, since the SLN hydrogel combined with MSB increases the inhibition of auto-oxidation of L-DOPA, it can be effective for cosmetic applications as a skin-lightening product.

###  Irritation test on the skin

 Formulations used for dermal delivery, in addition to the delivery of the drug to the skin, should not cause any significant irritation or side effects. Therefore, due to the topical application of MSB-SLN, it should be prevented from causing hypersensitivity and immunogenicity to have the necessary safety and be considered as a harmless production.^48^ A typical side effect caused by formulations prepared for dermal delivery is skin irritation, which is mostly related to the drug content.^48^ The score of erythema and edema (skin irritation) as a result of using MSB-SLN gel, MSB plain gel, blank SLN gel, and formalin is demonstrated in Table 2. According to Table 2, the average value of edema and erythema for MSB-SLN is 0.167, while for other samples and formalin is much higher than this value. On the other hand, Draize, Woodward, and Calvery previously stated that erythema and edema scores of 2 or less indicate that no skin irritation has occurred and are classified as negative. Therefore, according to the results, it can be said that MSB-SLN not only did not cause any skin irritation, but it is suitable as a safe formulation for skin application.

**Table 2:** Cutaneous sensitivity values after local application

**Rat **	**Control**		**MSB-SLN gel (F4)**		**MSB plain gel**		**Blank SLN gel**		**Formalin**	
	**Erythema**	**Edema**	**Erythema**	**Edema**	**Erythema**	**Edema**	**Erythema**	**Edema**	**Erythema**	**Edema**
1	0	0	0	0	0	1	1	1	4	3
2	0	0	0	0	1	0	0	0	3	3
3	0	0	0	0	1	1	1	1	4	3
4	0	0	0	0	1	1	1	1	4	3
5	0	0	0	0	0	1	1	0	3	3
6	0	0	1	1	2	2	0	0	3	3

A: Erythema score: 0, nothing; 1, minor; 2, well defined; 3, moderate; and 4, scar development. B: Edema scale: 0, none; 1, slight; 2, well defined; 3, moderate; and 4, severe. * Significant compared with formalin (*P* < 0.05).

## Conclusion

 Production of rhamnolipid biosurfactant was successfully done with *Pseudomonas aeruginosa* ATCC 27853 in a culture medium containing sunflower oil as a carbon source. Then, SLNs containing MSB (a hydrophilic drug) were successfully produced using a combination of this biosurfactant and a non-ionic surfactant. Solid state studies demonstrated that SLN components had no chemical interactions with the drug, which is in SLN in an amorphous form. The value of zeta potential, particle size, PDI, and an average of EE% for F4 formulation was -6.28 ± 0.33 mV, 117.26 ± 1.12 nm, 0.262 ± 0.002, and 83.34 ± 0.75%, respectively. In addition, the results of the MTT test and skin irritation showed no toxicity for HHF cells (due to a higher percentage of cell viability equivalent to 85.58 ± 1.77 %) and no skin irritation (due to edema and erythema score of 0.167) when treated with MSB-SLN. Also, according to the results of L-DOPA auto-oxidation inhibition and preventing synthesis of melanin studies, it was found that the anti-hyperpigmentation activity of MSB-SLN was much higher compared to bare SLN and simple MSB. In this research, it was shown that rhamnolipid-based MSB-SLN as an anti-hyperpigmentation formulation has the potential to be used and combined in cosmetic and pharmaceutical products.

## Acknowledgments

 The present research has been financially supported by the grant awarded by the investigation council of Babol Noshirvani University of Technology.

## Competing Interests

 The authors declare no conflict of interest.

## Ethical Approval

 All animal experiments were dependent on ARRIVE principles, the U.K. Animals (Scientific Procedures) Act 1986, and associated guidelines, EU Directive 2010/63/EU for animal experiments. All experimental and surgical protocols were assessed and approved by the animal and ethics review committee for Animal Experimentation at Mazandaran University of Medical Sciences (Protocol No. 9 of Sep. 28, 2021) Sari, Iran.
